# Postcolonial penality: Liberty and repression in the shadow of independence, India c. 1947

**DOI:** 10.1177/1362480615625762

**Published:** 2016-01-13

**Authors:** Mark Brown

**Affiliations:** University of Sheffield, UK

**Keywords:** Citizenship, colonial India, crime history, Criminal Tribes Act 1924, critical criminology, decolonization, indigenous justice, labelling, penality, post-colonial penality

## Abstract

This article reports primary archival data on the colonial penal history of British India and its reconfiguration into the postcolonial Indian state. It introduces criminologists to frameworks through which postcolonial scholars have sought to make sense of the continuities and discontinuities of rule across the colonial/postcolonial divide. The article examines the postcolonial life of one example of colonial penal power, known as the criminal tribes policy, under which more than three million Indian subjects of British rule were restricted in their movements, subject to a host of administrative rules and sometimes severe punishments, sequestered in settlements and limited in access to legal redress. It illustrates how at the birth of the postcolonial Indian state, encompassing visions of a liberal, unfettered and free life guaranteed in a new Constitution and charter of Fundamental Rights, freedom for some was to prove as elusive as citizens as it had been as subjects.

‘It is still unusual’, wrote the anthropologist Peter Pels (1997: 176), ‘for researchers to fully escape the dichotomy of colonial state and oppressed and/or resistant others, and to understand how much colonial empires were fragmented by other tensions’. In a literature replete with binaries—of metropole and colony, colonizer and colonized, elite and subaltern and, perhaps finally, colonial and postcolonial state — it is possible to imagine the actors so denoted should hold some emphatic power of perspective (taking, making, changing), giving force and definitive character to each side of the binary divide over which they are ordered. In this article I use a case study of the articulations of penal power in India in the years preceding and then immediately following independence in 1947 to examine the continuities and disruptions of governance over the colonial/postcolonial divide. For while the new postcolonial states of India and Pakistan were wracked by ethnic and religious violence at the very moment they gained their freedom, and while it is that violence and subsequent eruptions of nationalist passion that gain attention (such as the border wars fought between India and Pakistan, the dissolution of the bifurcated Pakistani state and the dispute over Kashmir), in penal governance terms at least the colonial/postcolonial divide was marked not by disruption but indeed by an impressive continuity of practice and purpose.

In considering how this came to be, the article seeks to develop the idea and carve out a space for the study of postcolonial penality. The absence of attention to this area might be added to the list of ‘criminological blind spots’ noticed by Katja Franko Aas (2007: 297) as the discipline elides major global and historical processes in favour of a kind of fetishization of the domestic now. This article’s engagement with India’s postcolonial moment will require connecting with a number of contemporary debates over the character of western intervention in what we now term the global South. Included here are questions of historical legacy, and particularly the question of whether contemporary political and international development challenges (corruption, patronage, big-man politics, sectarian/tribal looting of the state via participation in democratic politics, enduring tribal internecine warfare and so on) can be sheeted home to the legacies of colonialism. The temper of this debate is reflected in Joy Asongazoh Alemazung’s (2010: 65) argument in respect of Africa that ‘colonial impacts on post-colonial states in Africa’ must be understood ‘as colonial legacies. Some of these legacies include: neo-patrimonialism and clientelism, neo-colonialism (continuity in continuation of western control and dominance), authoritarianism, ethnic division and rivalry’ (see also Mamdani, 1996, later). Indeed, it is precisely the long shadow of colonialism, together with the nepotism of the ruling elites who replaced it, that for Alemazung (2010: 64) largely explains the preservation of Africa as ‘a poor and devastated region of the globe’. Colonialism can be reprised in other ways also. Abdullahi Ahmed An-Na’im (2013: 197), for instance, argues in this respect that ‘transitional justice scholarship and strategies are neocolonial because they view indigenous conceptions of justice as a distraction from the grand “modernizing” mission of North Atlantic societies’.

In engaging with these critiques of colonial rule this article examines the virtues and limitations of a particular vision and instance of colonialism, approached through the nested case studies of, first, British rule in India and, second, the governance of crime and marginal social orders across the colonial/postcolonial divide. It is therefore a study not so much of what colonialism ‘did’ but more so of what it changed and what it left behind. It is interested in what became possible and was thought desirable when the open horizon of postcolonial statehood lay before the new India’s governing classes. The article thus addresses directly the question of whether an anti-colonial movement born in the pursuit of freedom and liberty would be able to achieve the rupture in penal practices that such goals require and, if not, quite why.

The specific case to be examined here is the last years of what many view as a most egregious piece of colonial policy and legislation, the Criminal Tribes Act 1924, and its post-1947 replacement, but effectively also its rehabilitation and extension, within a new postcolonial grammar of habitual offender law. As the case study here will illustrate, the governmental project of managing these problematic individuals and communities spanned the artificial divide of colonial state and independent nation, where the latter was supposed to usher in a new, distinctive, postcolonial era. This era was conceived as a time in which, as Jawaharlal Nehru (1947) proposed in his famous *Tryst with destiny* speech on the eve of independence in August 1947, ‘India will awake to life and freedom […] when we step out from the old to the new, when an age ends […] the past is over and it is the future that beckons to us now.’ Quite why freedom for some was to prove as elusive as citizens as it had been as subjects is a question this article aims to address.

The remainder of the article is divided into five sections. The first sets out a theoretical framework within which the study of postcolonial penality might be grounded. It ranges from seminal work on Africa to the notion of postcolonial legalities and on to the more recent idea of the ‘everyday state’ as a way not only to problematize the binary of colonial versus postcolonial rule but so too to emphasize the precarious contingency of postcolonial citizenship. Section two will describe the history and character of the criminal tribes policy and amendments and extensions to its enabling legislation, bringing it to the point of the present case study in the late 1940s when, by various estimates, somewhere between three and 13 million Indians were subject to penal control and restricted access to law under its provisions.^1^ Section three presents the case of efforts in local and central government between 1937 and 1947 to amend or dispense with the Act and a detailed analysis of the 1951 *Report* of the Criminal Tribes Act Enquiry Committee, a body established in 1949 to investigate this instrument of colonial oppression developed within the pre-history of the Indian nation, or what Nehru (1947) described as the ‘period of ill fortune’. Here it will be observed that in fact far from dispensing with or dismantling this leg of the colonial penal apparatus, both local governments^2^ and the Enquiry Committee alike envisaged a need to expand its remit. Not only did they propose an almost identical apparatus for the new nation state, albeit with some finessing of language and recognition of the notionally changed political status of those who would fall within its purview, but they also chose to embrace it within the general body of law rather than to leave it, as colonial governments had, as a piece of extraordinary legislation. Section four returns to the notion of the everyday state as a potentially useful theoretical frame for understanding these features of postcolonial penality in India. Finally then, the article will conclude with some thoughts on how sense can be made of all this, reflecting upon the study’s implications in light of contemporary diagnoses that it is liberal institutions and visions that fragile, post-conflict and generally postcolonial states require in order to secure the safety, freedom and prosperity of their citizens.

## Postcolonial penality: Continuities, legalities and the ‘everyday state’

The question of what could be expected of postcolonial states is a vexed one. In the wake of the Second World War European powers that since the 17th century had slowly but progressively colonized swathes of Asia and Africa now began rapid decolonization. Many colonial regimes in Africa were still relatively new and European tenure there had been, to redeploy Hobbes’s famous epithet of life outside political community, nasty brutish and short. That many post-independence governments and leaders in Africa would turn out to be equally or even more savage and venal has given rise to intense debate as to how this could have come to pass. Alemazung (2010) and An-Na’im (2013) cited earlier both reflect elements of Mahmood Mamdani’s important thesis here. For Mamdani (1996: 285) it was the dual and oppositional structure of colonial governance and legal orders in Africa that set the seeds for postcolonial failure. Here, new states often ‘succumbed to caprice and terror on the morrow of independence’. Whether these new states sought to redress problems of urban/rural governance or to settle problems of rivalling ethnicities, either way, he argues, they tended to ‘soften one part of the legacy while exacerbating the other’ (Mamdani, 1996: 26). The result: considerable continuities in the oppressive character of rule over the colonial/postcolonial divide.

Outside Africa and with particular reference to the longer standing and undeniably more liberal models of governance employed on the Indian subcontinent, Upendra Baxi (2000: 541) has analysed the development of what he terms postcolonial legalities. His attention is drawn to constitutionalism as a defining feature of efforts of many postcolonial states to demarcate a kind of ‘historic rupture’ of independence. Thus, whereas Mamdani’s account emphasizes how new African states’ efforts to escape the colonial dispensation so often led to them immediately reproducing important parts of it, Baxi (2000: 542) suggests that postcolonial constitutions mark at least an attempted paradigm shift, experienced even as ontological rebirth, a claim ‘to self-determination not warranted by imperial legality’. Yet ‘pathologies of power’ (Baxi, 2000: 551) inherent within the practice of postcolonial constitutionalism tend also towards a reproduction of certain forms of class and social order. Here, newly independent elites quickly recuperate not only the visible trappings of colonialism but so too its visions and anxieties. ‘In many a society’, Baxi (2000: 551) argues, ‘the bulk and generality of postcolonial “citizens” are hapless victims of “governance” beyond the pale of accountability’. The law also and importantly seems much less amenable to the kind of ontological renovation sought in postcolonial constitutionalism itself. As Baxi observes and a number of other scholars have elaborated, colonial law’s repressive tendencies have been retained and even innovated upon within new post-independence discourses of security. Nasser Hussain (2003) has illustrated this with respect to the doctrine of emergency in British India, drawing attention to a colonial jurisprudence from within which postcolonial states’ repressions find support. Anil Kalhan (2010; Kalhan et al., 2006) has traced these claims to emergency powers and extra-constitutional rule through the modern history of India and Pakistan while, in a similar fashion, Jinee Lokaneeta (2011) has examined practices of torture in the new India. Delimitations upon rights, we are left to conclude, are not solely the preserve of the colonial state.

Yet for as much as the work of Mamdani, Baxi and others brings us closer to recognizing the inherent limitations of postcolonial statehood it is relatively silent on one important matter. This is the question as to how postcolonial states’ mimetic tendency—a tendency towards ‘the reproduction, with a range of variation’ of discourses and practices of colonial governance (Baxi, 2000: 544)—singles out particular individuals or groups for such treatment and the form of the settlements reached thereafter. In the last decade or so, however, a small literature has begun to emerge tracing the fractures that separated these grand narratives of postcolonial statehood from the lived experiences of individuals who, to use the title of Sherman et al.’s (2014) recent collection, had been overnight transformed *From Subjects to Citizens*. Their collection is part of this wider attempt to examine what is termed the ‘everyday state’ (see also Fuller and Bénéi, 2001; White, 2013), which they describe as ‘the various processes by which elite ideologies and institutions are interpreted, translated and manipulated at the quotidian level by men and women as they negotiate their lives’ (Sherman et al., 2014: 1).

One example of this approach is Eleanor Newbigin’s (2014) study of the way Hindu and Muslim communities in colonial and postcolonial India negotiated the shape and parameters of the personal religious laws that should apply to them. While the rights concerned here are clearly of a different order to those of individuals or groups subject to security measures, the example is interesting for it reminds us that not all power is repressive and nor is its articulation beyond the influence of actors whether subaltern or elite, citizens or subjects. Since colonial government had from its earliest times marked out the private domestic and religious sphere as a domain of non-interference (see Duncan and Derrett, 1961; Lariviere, 1989), much of the structure of rights and liberties that attached to individual lives, for men versus women, individuals versus families, religious versus civil tribunals and so on, were determined in this process of negotiating the scope of personal laws. That in the postcolonial period different religious communities continued to be governed by different personal laws, laws that distributed rights in markedly different ways, attests to the way in which the supposedly universalizing principles of secular citizenship reflected in the 1950 Constitution’s Fundamental Rights were always mediated at the point of practical connection between the state and its various citizen-subjects and their equally various communities.

Much of the everyday state literature therefore points towards this mediated feature of citizenship in the postcolonial state. Even if new postcolonial states represented themselves in terms of abstract principles, as embodied in the Indian Fundamental Rights, in practice there were both strong continuities across the colonial/postcolonial divide and strong tendencies for the state to view its subjects in communal terms. On this point Newbigin (2014: 37) makes a ‘plea’ for greater recognition ‘that 15 August, 1947 did not mark a *tabula rasa* in Indians’ relationship with the state, but that people’s views of and relationship to notions of citizenship were deeply shaped by pre-existing debates and social structures’. It is to some of those pre-existing debates and structures that I now turn.

## Penal power and colonial rule: The Criminal Tribes Acts

Critics of empire have tended to grasp at bold instances of colonial violence in their analyses of colonial rule. In the Indian context, events attracting attention have included the Thuggee campaign of the 1830s (see Sleeman, 1836; Wagner, 2009), or the notorious Jallianwala Bagh massacre of 1919 (Collett, 2006). The criminal tribes policy is much less well known, but its operation spanned the years from 1871 until after independence and its reach, taking in first hundreds, then thousands, then millions of Indians, was as impressive as the draconian limitations upon personal freedom and movement it provided for.^3^ It thus stands as a useful example of the routine and increasingly anodyne measures of illiberal control that were at once peripheral yet also indispensible to colonial governance and that, presumably, would quickly be removed under a new, independent, postcolonial dispensation. Indeed, no lesser figure than Jawaharlal Nehru himself had singled out the Criminal Tribes Act for particular mention, describing in a 1936 speech the Act’s ‘monstrous provisions’ as ‘a negation of civil liberty […] out of consonance with all civilised principles’ (cited in D’Souza, 2001: 57).

### The Act and its targets

The Act was originally directed at a combination of itinerant communities who, in their travels, were thought also to dabble in various sorts of petty crime, as well as certain settled communities that, on the other hand, frequently decamped on what were believed to be thieving expeditions. Between them these groups created particular problems of internal cross-border policing. Measures to prosecute and punish them existed under the Indian Penal Code 1860 and Code of Criminal Procedure 1861, but they were seldom used due to the difficulty of gathering sufficient evidence of the supposed misconduct. Acute pressure therefore developed during the late 1860s for more easily accessed powers to restrict movement, force settlement and induce tribes and communities to take up sedentary agricultural forms of life and livelihood. Most of the tribes and communities concerned were either outcast or very low in the social hierarchy and so the problems they posed can be understood also in terms of the problems of governing the social margins of Indian society.

The origins of the criminal tribes policy lie in a complex set of local initiatives developed in the Punjab and North Western Provinces (NWP) in the early 1850s. A complete genealogy of the policy is beyond the scope of this article, though I have attempted as much in my book *Penal Power and Colonial Rule* (Brown, 2014). Nevertheless, four features of it are worthy of brief note. First, the criminal tribe concept itself emerged in the late 1840s and early 1850s but was, in its early incarnations at least, little distinguishable from accounts of tribal or hereditary conduct then circulating in Britain and elsewhere. Henry Mayhew’s chronicle of British urban life *London Labour and the London Poor*, for example, includes descriptions of ‘costermongering [which] seems an hereditary pursuit’ (1851: 479). What changed after mid-century was the sense that, in India, tribes’ conduct was determined not *only* by social forces, creating a distinct criminal habitus, but that two further, parallel, forces—of religion and caste—worked to make crime an occupation not only sanctioned but in a sense also required of the tribesman. Such forces were interpreted to mean that for the hereditary Indian criminal ‘it is his trade, his caste, I may say his religion to commit crime’.^4^

A second point to note is that the problems such tribes were felt to pose to colonial government were marked not by their particular seriousness so much as by their intractability and three related challenges that this threw up. To begin, tribes tended to be highly mobile at a moment when sedentarization was at the centre of colonial policy. They also posed problems of identification, in the sense that not only were they mobile but they seemed to the colonial eye indistinguishable from the great mass of India’s lower orders, most of whom were not criminal at all. Finally, the tribes’ criminal conduct was interpreted as a rejection of agricultural endeavour, which was felt to be the heart and engine of native life, and so an affront to the peaceful and ordered society British government sought to fashion on the subcontinent.

A third observation is that despite the second half of the 19th century being generally associated with a resurgent and, in the jurist JF Stephen’s (1883) words, a ‘belligerent’ British imperialism, a great weight of opinion within Indian administration in 1870 held that measures of punitive control and restrictive surveillance were unsuited to the purpose and spirit of British rule. For the Bombay Government^5^ such measures were ‘objectionable’ as too for the Chief Commissioner of the Central Provinces^6^ such a system would ‘be unnecessary’ in practice ‘as well as objectionable in principle’. Even in the North Western Provinces,^7^ one of the areas where criminal tribes were to be targeted, they were viewed as ‘vexatious surveillance’ and to ‘savour more of the age of barbarism and are opposed to all ideas of humanity and civilization’.^8^

Despite such misgivings the Criminal Tribes Act was passed in the Legislative Council and received the assent of the Governor General on 12 October 1871. The Act in this first incarnation had six distinctive features. First and most generally, it rode above the normal penal law, prescribing restrictions and setting punishments for acts that in most cases did not constitute criminal offences. Second, it applied to whole tribes that a local government (initially, Punjab, NWP and Oude) would claim to be ‘addicted to the systematic commission of non-bailable offences’ (s. 2), providing for their notification as a ‘criminal tribe’. Third, any tribe, gang or class so notified could be subjected to surveillance measures such as roll calls, restricted movement, a passport system and a range of other disabilities. Infraction of these rules was subject to administrative punishments, including whipping. Settled tribes could be moved to a new locality and nomadic tribes settled down, with provision for reformatory settlements to be established for recalcitrant members of both. Fourth, there would be no access to legal review of decisions, in that ‘every such notification shall be conclusive proof that the provisions of this Act are applicable to the tribe, gang or class specified therein’ (s. 6). Fifth, where a criminal offence was committed by a registered person sentence would be magnified, such that a second conviction for one of the scheduled offences would result in a mandatory seven years’ imprisonment; a third offence in transportation for life. Finally, the Act sought to responsibilize village headmen and landowners in the surveillance programme, making them accountable for monitoring comings and goings of registered persons and liable to punishment if they failed to do so.

The final observation to be made on the criminal tribes policy at this point concerns its effectiveness and early administration. Initially local governments struggled to make the case to the Government of India that their troublesome tribes did in fact meet the criteria for notification. Many anecdotes of tribes’ criminality abounded, but firm evidence was in fact scarce. As the NWP Legal Department noted on one draft application in 1872 prior to its being sent on to Calcutta:

It would, I think, have been more satisfactory if some closer description of their recent doings had been given than that they are always heard of as professional thieves, that they openly boast of having been trained from infancy in pilfering, and that they have ‘almost denuded the neighbouring villages of sheep and goats’ and that on every opportunity they will steal […] But for any thing more specific the reports go back to 1853 and to 1863 before the colony was settled at Bidowlee.^9^

The severity of the Act’s provisions combined with scant resourcing for its administration initially left it for the most part ineffective. Yet that ineffectiveness was sheeted home not to bad design or maladministration but, surprisingly, to leniency, such that in 1896 a new Bill was put forward to strengthen the Act. While recognizing the severity of existing provisions, the new Bill proposed that ‘the altogether exceptional circumstances of the case’ were of such a quality

as to justify still more drastic measures in order to strengthen the hands of the Government in coercing and, if possible, reforming the members, and more especially the rising generation, of such tribes, composed as they in fact are of criminals of the worst type, whose only occupation is crime.^10^

This amending legislation was passed, but following a partial review of the policy by the Indian Police Commission of 1902 (IPC, 1905) new legislation to provide all-India application of the Act and to apply it to a wider range of criminal groups was put forward. The Criminal Tribes Act 1911 repealed all earlier legislation and gave effect to these expansive objectives. But again first in 1923 and then substantively in 1924 new legislation again widened the reach of the Act, making provision, for example, for troublesome groups to be deported from the native states into British jurisdiction whereupon they could be notified as criminal tribes and then once again deported within India to provide labour for industries such as the tea plantations (see generally Kamat, 2010; Radhakrishna, 2001). It was in this form that the criminal tribes policy finally settled, became mainstreamed and developed into a behemoth of illiberal control, surveillance and punishment.

### Who were the criminal tribes?

Stated in this way the question of the criminal tribes’ identity is twofold: who were they as a class or group, a section of Indian society; and who were they as people? The task and hope of excavating individual lives and subjectivities from the colonial archive is probably misplaced. As Gayatri Spivak (1985: 270) observed in her essay ‘The Rani of Sirmur’, the colonial subject—even one as important as the wife of a Raja—‘emerges only when she is needed in the space of imperial [knowledge] production’. Even then, most remain nameless, as did Spivak’s Rani. In the later period of British rule the existence of petitions, participation in organized labour and the like provide some keys to tribes people’s perceptions and aspirations (see Kamat, 2010). In terms of the founding moments of the policy, which is the concern here, insights are speculative and refracted through the official archive, but they are not completely absent and will be returned to in a moment.

As a class we are on firmer ground. Those designated criminal tribes tended to belong either to marginal social orders on the fringes of sedentary Indian society for whom petty crime was a matter of subsistence—such as Sansis; or to peripatetic groups who criss-crossed India trading salt or other goods from bullock and donkey, but whose lifestyle became increasingly precarious in a rapidly modernizing society—such as Koravas; or to hill and forest tribes, whose incursions upon lowlanders were less and less tolerated—such as Bhils;^11^ or to tribes whose number were scattered across wide areas of territory and might, in certain areas, have been settled agriculturalists, while in others they were thought to be criminal, roving and in the colonial argot, ‘predatory’—such as Minas. These, then, were the kinds of tribes targeted by the Act. But it is important also to note that the very same sorts of groups would later be targeted by the postcolonial state as it sought to govern the new nation’s social margins. The transfer of power in 1947 thus did little to release the state’s grip upon its marginal orders.

Who then were these people as people? It is from colonial servants’ interactions with them that some picture of the tribesmen and their lives emerges.^12^ In the 1857 uprising, groups who would later be deemed habitually criminal were recorded as giving good and faithful service in Rajputana where they saved many British lives and shored up defences against the so-called mutineers (Brown, 2014: ch. 4). Generally speaking, military officers seemed to view tribesmen as martial in character and official recruitment manuals spoke highly of them as individuals and soldiers. The Bhils, mentioned above, were described in Bonarjee’s *Handbook of the Fighting Races of India* as being ‘[c]hief among the aboriginal soldierly tribes’ (1899: 142). Their contact with British authority had begun in 1817 when a ‘well-meant but rather unwise attempt’ was made ‘to prematurely interfere with the rights the Bhils had enjoyed from time immemorial, to levy blackmail on all who wished to be safe from their depredations’ (Bonarjee, 1899: 144). In another recruiting manual, Bingley’s *Notes on the Warlike Races of India and Its Frontiers*, the Mina was described as ‘a smart, faithful, and obedient soldier’ (1897: 42).^13^ Summing up the position of these tribes in the mid-1870s, Lieutenant Colonel WH Beynon, Agent to the Governor General in Rajputana, reported that ‘[t]he unruliness and predatory habits of the Bheels and Meenas are closely connected with the injustice, if not the cruelty, which they have constantly experienced at the hands of the [Native] State officials and the ruling castes’.^14^ Where they did secure a measure of protection from feudal landlords it often came at a cost: as one annual report noted, ‘the local landholders, who should control them, still keep them in pay, and share their booty’.^15^ They would find, however, that in coming to the attention of British authority the difficulties of life were not necessarily ameliorated and the changes of habitus expected of them often were not to their liking. As one frustrated administrator was to remark, ‘[t]hey have an almost invincible repugnance to agriculture’.^16^ Indeed, the failure of many tribes placed in reformatory villages to grow enough food for their own sustenance meant they were often only ‘kept from starving by direct grants from Government’.^17^ Such were the difficult circumstances under which the criminal tribes policy was born.

## The Criminal Tribes Act in the shadow of independence

The criminal tribes policy had been given effect through central Government of India legislation, the final version being the Criminal Tribes Act 1924. Yet in a curious double movement, the Act gave local governments concurrent jurisdiction to determine whether or not it should apply in their territories and, if necessary, how it might be amended to reflect local needs and conditions. By the late 1930s there was growing sentiment that the Act was outmoded and inconsistent with the liberal freedoms that Indians, albeit still colonial subjects, should enjoy. A Criminal Tribes Act Enquiry Committee appointed by the Bombay Government in 1937 recommended significant dilution of the punitive elements of the legislation and in 1942 the government there amended the Act accordingly. In Madras and Uttar Pradesh also reviews were undertaken around this time with a view to determining the appropriateness of the Act’s restrictions and punishments to the modern day. Madras amended the Act as it applied in its territory twice, once in 1943 and again in 1945. By 1946 a second Uttar Pradesh enquiry committee was referring to criminal tribes as ‘habitual offenders and vagrants’ and recommending new legislation framed in these terms rather than the now anachronistic idea of Indians labelled criminal by birth.

At the level of central government, private members bills seeking repeal of the Criminal Tribes Act were introduced in 1946, 1947 and 1949. The first of these was never properly pursued and fell by the wayside. In 1947 a repeal Bill was introduced on 6 February by Sri R Venkatasubba Reddiar—barely six months before independence—but the demand for repeal was withdrawn when he was informed that provinces themselves were beginning to take decisive action and that, indeed, ‘the Madras government now has before its Legislature a Bill to proceed with the repeal of the Act as far as that province is concerned’.^18^ Similarly, in 1949 the demand for repeal was withdrawn based upon a promise made by the Minister for Home Affairs that a central government enquiry committee would be appointed to review the Act at an all-India level. The Minister made good upon his word and on 28 September 1949 a Criminal Tribes Act Enquiry Committee was appointed with a remit to consider the legislation in all its aspects and make suitable recommendations for repeal or reform.

### ‘This free land of ours’: The Criminal Tribes Act Enquiry Committee deliberations

Following its first meeting in December 1949 the Criminal Tribes Act Enquiry Committee developed a questionnaire which it sent to 300 relevant members of local governments and interested parties. In the course of its enquiries it interviewed more than 200 witnesses, toured the Punjab, Uttar Pradesh, Bihar, West Bengal, Orissa, Bombay and Madras. Its members travelled more than 10,000 miles by train and an estimated 1200 miles by road, visiting along the way five settlements in which criminal tribes were interned and 14 colonies or villages of a reformatory character where they had been placed.

‘Wherever we went’, the Committee reported, ‘we heard one single cry from all the criminal tribes that whereas India obtained freedom, they continued to be in bondage and their demand for setting them free by repealing the Act was insistent’ (Criminal Tribes Act Enquiry Committee, 1951: 81; hereafter, Enquiry Committee). More prosaically, the Committee determined that the Act’s classification of individuals as members of a criminal tribe by dint of birth was almost certainly unconstitutional, observing ‘that no one in this free land of ours should be treated as a criminal merely because of the incident of birth’ (Enquiry Committee, 1951: 91). Offensive to the Constitution also was the compulsory work required of registered tribesmen in reformatory settlements, which not only constituted an offence under the Indian Penal Code, but was also opposed to Article 23 of the Constitution and placed India in breach of the International Labour Organization’s (ILO) Convention on Forced or Compulsory Labour. Indeed, the Ministry of Labour gave evidence that it was due only to this Act that India was unable to become a signatory to the ILO Convention. At the provincial level, the Act had been repealed in Madras in 1947 and Bombay in 1949. In Uttar Pradesh the local government was moving towards repeal of the Act based upon pre-independence recommendations. And in the former native states of Rajputana new legislation to repeal and replace the Criminal Tribes Act had been passed in February 1950. The case for doing away with the criminal tribes policy could not seem to be stronger.

### ‘Habitual offenders and vagrants’: Towards a new grammar of control

Upon the abhorrence of a system whereby children born into certain social groups came automatically to be classified as members of a criminal tribe there was no doubt or debate. Past this point, however, the Enquiry Committee soon discovered strong demands for continuity of practice with the former colonial approach. The taint of criminality by birth must be done away with, but the idea that the conduct of some of these groups’ members—both in terms of their criminality and their nomadic, wandering, instincts—required a firm and distinctive architecture of suppression, and where possible reform, was widely held. Wherever the Criminal Tribes Act had been repealed it had been replaced by habitual offender statutes,^19^ but as the Enquiry Committee observed, in many respects these new statutes merely replicated the Act minus one or two of its more offensive clauses.

When local governments and their key officials were asked to comment upon the desirability of retaining the Act or repealing it, repeal was in the main desired. But respondents almost unanimously made the caveat that suitable habitual offender legislation to achieve the same effect, but without the vices noted above, should be developed and should be enacted so as to overlap in temporal terms with the repeal process. What is most striking in these responses is the continuity of thinking among those engaged in criminal tribes management and in government more generally with rationalities and practices running right back to the turn of the century. Surveillance and suppression concerns raised by the Indian Police Commission of 1902 returned to the page in the responses of local governments in the immediate post-independence period. Themes that are today mainly associated with the despotisms of European colonial powers, particularly around the identification, pacification and sedentarization of native subjects, were strongly presented by postcolonial state officials not just as desirable but indeed as indispensible. The Deputy Commissioner for Criminal Tribes in the Punjab, for example, reprised in his submission the idea of roundups and enclosures that had been a feature of criminal tribes administration in the years before 1920, arguing that ‘[g]enerally speaking persons who wander about start as beggars and end as hardened criminals’, so that ‘[a]ll over the country simultaneous raids must be carried out to catch the vagrants and then settle them on useful occupations’ (Enquiry Committee, 1951: 88).

Many echoes were also heard of the suppositions about tribes’ behaviour that had led to the Act’s initial passage in 1871. And now just as before, they were generally unsupported by any data. The Criminal Tribes Officer in Gwailor gave the tone of these when he proposed of the Kanjar tribe that ‘[o]ur suspicion is that [they] commit crimes and remain undetected and hence the whole tribe of Kanjars must be considered as habitual offenders’ (Enquiry Committee, 1951: 87). In other cases the Act’s retention was felt appropriate given the myriad other claims upon government time and attention in the post-independence period. The Government of Orissa, for example, proposed that ‘the Act should remain in force for the present and the matter should be reviewed after 10 years’ (Enquiry Committee, 1951: 84), while the government of the new state of Madhya Bharat informed the Enquiry Committee that ‘[p]remature abolition of the old system of control may lead to results with which our weak administration may find it difficult to cope’ (Enquiry Committee, 1951: 87).

For its part the Enquiry Committee drew two primary conclusions from responses to their 300 solicited questionnaires and something like 200 interviews undertaken with stakeholders. First, it recognized widespread consent on the need to shift offender classification from a communal to an individual basis and to replace the idea of criminal tribes with that of habitual offenders:

After careful consideration, we have unanimously reached the conclusion that the time has arrived, if it is not already overdue, for the replacement of the existing Act by a Central legislation applicable to all habitual offenders without any distinction based on caste, creed or birth.

(Enquiry Committee, 1951: 90)

At the same time, however, the Enquiry Committee noted many reservations with the habitual offender statutes already enacted in provinces like Madras and Bombay. Rather remarkably, the Committee tended to view the new legislation as having swung too far in the direction of freedom and indeed favoured a move back towards the more deeply intrusive control apparatus of the colonial era.

### ‘Not legally possible under the ordinary criminal law’: The need for extraordinary measures

It had been a central argument of JF Stephen, the law member who shepherded the Criminal Tribes legislation through the Governor General’s Legislative Council in 1870–1871, that ordinary criminal law was too weak to deal with the threat posed by criminal tribes. Introducing the Bill on 3 October 1870 he proposed that part of the problem lay in the fact ‘that English lawyers and law-courts had a most exaggerated estimate of the power of the ordinary criminal law to cope with organized crime’. Indeed, he suggested, ‘[t]o suppose that ordinary processes of law would ever put down crime was like supposing that sportsmen would exterminate game’.^20^ Now, as representatives of the postcolonial state came to look at the problem, they tended to agree. The key to heading off the threat posed by members of these marginal social groups, the Enquiry Committee (1951: 91) observed, was an extensive system of surveillance whether or not those so targeted had committed any offences: ‘but’, they observed, ‘this is not legally possible under the ordinary criminal law’.

Furthermore, despite the mooted shift from a communal model of classification, grounded in concepts of tribe, class or gang, to an individual model based upon the idea of habituality, there was the problem that India remained, in practice, a society very much ordered by those very communal groupings an individualizing Habitual Offenders Act would seek to extinguish. Where groups were nomadic, peripatetic, bodies or where the habitual offenders were serious offenders there would be a need to restrict their movement, to corral them and confine them in the old colonial fashion. But again it was observed that there were ‘also no provisions under the existing criminal law for establishment of settlements, where attempts can be made at reformation of these offenders and also for the proper treatment of their children’ (Enquiry Committee, 1951: 91). Gradually, step-by-step, the Enquiry Committee began rebuilding the machinery of colonial control.

In discussing the children of the criminal tribes the Enquiry Committee had earlier registered its wholesale support for the conclusions of the Indian Jails Committee of 1919–1920, a body that had looked askance at the criminal tribes policy and made strong recommendations, unheeded, for its incorporation into the normal machinery of justice and its governance under ordinary principles of individual rights and social equity. The Jails Committee had described the Act’s removal of children from parents at four years of age as ‘an act of inhumanity which it would be hard to justify except on grounds of unavoidable necessity’ and, presciently, warned of the settlements system that ‘it is very important that they should not be allowed to degenerate into a novel type of jail where members of the criminal tribes can be locked up indefinitely without the usual formalities of a trial’ (Indian Jails Committee, 1920: 327, 331). But now the Enquiry Committee discerned just those very ‘grounds of unavoidable necessity’ to be at hand: ‘[w]e further suggest that in the proposed Act the State Governments be empowered to order the segregation of the children of habitual offenders from their parents’ (Enquiry Committee, 1951: 99). A list of ‘circumstances’, essentially describing the precarious conditions of life experienced by many criminal tribes on the margins of Indian society, provided the justificatory criteria for children’s removal.

Thus it was that in the immediate postcolonial moment and at the very point where Nehru’s (1947) vision of moving ‘from the old to the new, when an age ends […] [and] the past is over’, the Criminal Tribes Act Enquiry Committee first deconstructed and then piece by piece reconstructed an unambiguously illiberal vision of penal control for subordinate social classes. The window dressing changes of nomenclature, from criminal tribes to habitual offenders, does little to change the fact that for some citizens of the new India ‘freedom’ would entail inhabiting a much truncated version of universal liberal rights-bearing citizenship. How can we make sense of this?

## Liberty and the seductions of repression: Penality and postcolonial citizenship

Penal theory offers few guides to how mechanisms of control in postcolonial states might be understood. Some time ago I looked at the other side of the coin, suggesting that developments in contemporary western penality linking certain groups, and particularly sex offenders, within a subordinate form of citizenship might be understood as reprising a distinctly colonial logic (Brown, 2005a, 2005b; see also Hamilton, 2011; Moore, 2014). Viewed now, we might say the presumption that repression was the preserve of colonial regimes was premature. The case of these tribes deemed criminal within the arc of colonial penal power, yet quickly re-encompassed and reframed as habitual offenders under the postcolonial dispensation, points to a need for a more complex theoretical palette than penal theory currently has to offer.

One useful approach may lie within that trajectory of postcolonial theory I sketched in section one. It will be recalled that this body of work problematizes the presumption that independence movements founded upon claims to liberty, independence and freedom from the colonial yoke would, or perhaps could, deliver upon those ideals, instantiating them in the character and form of the postcolonial state. My inclination to draw upon the ‘everyday state’ literature lay in its focus upon citizen–state relations as subject to constant contest and negotiation. From this view, postcolonial citizenship emerges as a negotiated settlement between the state and a variety of classes of what might be termed ‘potential citizens’: individuals and groups within the territorial confines of new states at the moment of their birth (Sherman, 2014) who must in some manner be incorporated into the new nation.

One obvious site where such negotiation might have taken place was the extensive meetings, visits, deliberations and research of the new India’s Criminal Tribes Act Enquiry Committee. Understanding the fundamentally illiberal attitude of the Enquiry Committee towards one such class of Indian citizens is important here, since these were the first jousts in what would turn out to be a long and difficult negotiation between members of these tribes and the state of India over their status within the Republic.^21^ To achieve such understanding certainly requires recognition of the ‘pre-existing debates and social structures’ to which Newbigin (2014: 37) refers. But more than that, it also demands a critical appreciation of the material realities of work in the postcolony. And so too of the degree to which postcolonial actors often were no better prepared, and sometimes worse so, than their colonial predecessors for the task of giving effect to transcendent constitutional ideals of equality and freedom.

There is not space here to consider these problems in detail and the preceding analysis of the Enquiry Committee’s *Report* will leave no doubt as to the restricted vision of tribes’ citizenship status Committee members seem to have held. Nevertheless, four issues may be noted as bearing in material ways upon the field of potential outcomes available to the negotiation process: the framing of different enquiry committees’ terms of reference; the body of knowledge underpinning decision making; the perceived limits of repression within a liberal order; and the presence, recognition and representation of subaltern voices.

### Administrative continuities and precedents

The first two of these issues reinforce the ‘everyday state’ literature’s observations of substantial administrative continuities over the colonial/postcolonial divide. Enquiry committees had been established in Bombay, Madras and Uttar Pradesh beginning in the mid-1930s, resulting in major changes to the Act’s operation even prior to India’s independence in August 1947. Looking at the terms of reference for these enquiries reveals there was nothing distinctively postcolonial about the terms set by the Ministry of Home Affairs in September 1949. Nor indeed was there anything distinctively postcolonial in the responses of local government officers, most of whom as Indians were filling the same positions as had existed under the British Government of India. Many of these officers had spent a lifetime within the cultural habitus of colonial police and social welfare bureaucracies and some would have contributed to the administration reports upon which the Enquiry Committee relied. Further still, these civil servants would have been aware that there was precedent, albeit limited, for the extension of criminal tribes mechanisms to individuals, both in the Punjab (see below) and Burma. ^22^ With respect to the character of the tribes under judgement—who they were as people, as new citizens—the knowledge upon which the 1949–1950 Enquiry Committee relied was almost entirely colonial in its derivation. Almost 140 pages of its *Report* were given over to descriptions of tribes’ habits, manners and offending proclivities culled largely from colonial policing and administration manuals, some of which dated well back into the 19th century. To the now-contemporary observer this might seem manifestly and massively inadequate, but in the immediate shadow of independence, what other kind of knowledge of so many of India’s inferior and marginal social orders could have been drawn upon?

### The limits of repression within a liberal constitutional order

More substantively important, however, is the governmental attitude to illiberal legislation. Remarkably, the Enquiry Committee’s *Report* evinced far greater tolerance for derogation from the kinds of citizenship rights promised free subjects under the Indian Constitution than colonial administrators were, for their part, prepared to countenance for their subjects. Indeed, what marked the criminal tribes policy out during the colonial era was not just its scope or its repressive character. Most important perhaps, though this has been little noticed in the literature, was its status as a piece of extraordinary legislation. In amending the Act in 1923, for example, the Government of India had expressly rejected the Indian Jails Committee’s (1920) recommendation that it should be brought within the normal laws and rules of India. Indeed, the Secretary of State for India in London had been at pains to clearly and unambiguously describe its special status. Referring to the 1918 Punjab Habitual Offenders Bill that replicated the criminal tribes machinery, he proposed that the restriction of movement and extension of police surveillance to individuals not yet convicted of any offence ‘accepts a principle which has not, I think, found a place in the permanent, as opposed to emergency, legislation of British India’. Referring to Bengal Regulation III of 1818, which provided for the preventive detention of political suspects without trial, as well as similar regulations in Madras and Bombay, he put them all together as measures of an exceptional character: examples of ‘exceptional legislation’ the passage of which ‘should not in itself be taken as a precedent for similar enactments in other provinces’.^23^

What is striking in the Enquiry Committee’s deliberations is the lack of any equivalent reference back to overarching ideals, either to the new Constitution’s meaning and vision or more generally to the liberal political ideals of freedom and civic participation. Alternatively, there might have been some effort to seek justification in necessity or discourses of security, as the work of Kalhan (2010; Kalhan et al., 2006) might lead us to expect. The demands of social order were implicit yet never reflected upon. That the Enquiry Committee seemed to imagine a form of secondary citizenship as so natural as not to require comment does, however, chime with Newbigin’s (2014) observations on Hindu agitation in respect of personal laws. ‘On closer inspection’, she concludes, it is clear that ‘Hindu reformers were not calling for freedom and rights for everyone’ (Newbigin, 2014: 12). If modern rights and freedoms constituted forms of power, then in a hierarchical society why indeed should they not be distributed hierarchically?

#### Can the subaltern speak?

Among the most withering criticisms of colonial rule in India has been that which emerged from the subaltern studies collective (see Chaturvedi, 2000; Ludden, 2002). In particular, questions relating to Indians’ voices, represented most famously in Gayatri Spivak’s (1988) rhetorical question and essay ‘Can the subaltern speak?’ Spivak’s point there and in the essay ‘The Rani of Sirmur’ (1985), discussed earlier, concerned the limited role Indian women’s voices (subordinated to colonial and patriarchal authority) held in shaping debate and resolving ‘the problem’ their conduct posed. For subalternists and many other postcolonialists, the failure to make space for ‘native’ voices in the records of empire is a kind of stake to be driven through the colonial heart, illustrating once and for all its malign character.

Yet from the evidence at hand here, the Enquiry Committee achieved little better. It visited a handful of criminal tribes settlements and villages on its long tour of India during 1949–1950. It is apparent from the appendices to its *Report* that members of criminal tribes were interviewed. But only five of the 127 tribes identified by the Committee were entertained to speak. And even then it is uncertain what kind of input they might have had. In many cases, like Spivak’s Rani, not even their names were considered worth noting: ‘Deputation of 4 Baurias’, or ‘Deputation of 20 Sansis at Dina Nagar Railway Crossing’ (Enquiry Committee, 1951: 126). What therefore stands out in the *Report* and what connects that document with much of the earlier colonial administrative literature reviewing the criminal tribes policy (e.g. Kaul and Tomkins, 1914) is precisely this absence of voices of criminal tribesmen and women themselves. It is as if the Enquiry Committee were unable to reconfigure the colonial cognitive horizon within which, for reasons both material and constitutive, they were caught. Brought together with the literature of the ‘everyday state’, emphasizing as it does the complex, contested and contingent nature of citizenship in the postcolonial state, this raises important questions about how the voices of subaltern groups in India and elsewhere might be rendered audible. The Enquiry Committee noted the complaints of tribes ‘that whereas India obtained freedom, they continued to be in bondage’ (Enquiry Committee, 1951: 81), but beyond this single instance within the *Report* there is no contribution of tribes people to the discourse of postcolonial restriction and surveillance.

Of course this is not to say that subaltern voices were nowhere to be heard at all. Indeed, debate on the criminal tribes and habitual offender provisions can be found in the then contemporary press and throughout the period of the Act’s operation aggrieved tribes people petitioned government on a variety of matters. But as the concern in this article has been with discourses of penal governance we are forced to conclude that subaltern voices penetrated little into these networks of power. Thus, in contrast to Newbigin’s higher caste Hindu males who successfully renegotiated the writ of personal laws, the criminal tribesmen and women were negligible and that form of reduction is apparent not only in the *Report* but so too in the new legislation that would soon follow it.

## Conclusion

This article has sought to develop the idea and indicate the importance of postcolonial penality. Through its Indian case study it has also contributed to what Sherman et al. (2014: 3) bemoan as the too ‘little discussion’ in contemporary scholarship of ‘the everyday aspects of the early post-1947 state [in India] or linked notions of citizenship-in-the-making’. It has contributed further evidence of what they term the ‘powerful continuities between the pre- and post-independence periods’ in terms of state function and lives lived in India. The article has shown how major reviews of a most odious example of colonial era penal legislation, the Criminal Tribes Act 1924, were undertaken across the colonial/postcolonial divide, spanning the years 1937–1952 and the emergence of India as an independent nation state in August 1947. In the final, post-independence, review that has sat at the centre of this article the colonial armature of control was first deconstructed on grounds of principle and offence, before being slowly rebuilt to satisfy the demands of local governments for continuity of rule and repressive control over those new citizens who sat on the margins of Indian society. Time and again it was noted that the ordinary criminal law could not be relied upon to address the special problems that criminal tribes—now relabelled habituals and vagrants—would pose to the new nation state.

This armature of control has been described here primarily in terms of continuity, but it is worth observing that at a number of points—from decisions about the removal of children from parents, to the incorporation of habitual offender measures into the ordinary machinery of law—the postcolonial state went farther than its colonial predecessor had been willing to go. The postcolonial reformulation of colonial modes was thus in many ways more repressive than what came before it. Similarly, with respect to the idea of a colonial/postcolonial divide, there is evidence that the continuities observed here were established much earlier than the ‘rupture’ point of 1947. From at least the 1920s and certainly by the 1930s the wind-down of the British colonial state had transferred significant political power into Indian hands at both local and regional levels. This is well illustrated in Manjiri Kamat’s (2010) study of criminal tribes’ involvement in labour disputes in the tea industry during the 1930s. Her study of criminal tribes’ experiences under the newly installed Congress administration there illustrates just how much that government moved within the same cognitive and strategic horizons as earlier British administrations.

In making visible these continuities in their various forms the article has therefore attempted to problematize any easy binary separation of colonial and postcolonial states and the governance thereof. In doing so, however, it has also raised a series of questions about the scope and concerns of postcolonial penality as a distinctive field of study. After all, what is it about the postcolonial state that is distinctive if not the ‘post’ of coloniality itself? In seeking to answer that in relation to the criminal tribes case study I have tried to tie a series of criminological questions to the wider theoretical horizon of postcolonial studies. Two ideas in particular—the notions of postcolonial legalities and postcolonial citizenships negotiated at the level of the everyday state—appear to have resonance for this work. Perhaps drawing them into some kind of resolution to the criminal tribes question might provide a pointer towards the value of studying postcolonial penalities.

One thing appears quite clearly from the detailed analysis of the criminal tribes policy’s postcolonial life reported here. It is that Indians’ capacity to claim or negotiate important citizenship rights in the critical founding moments of the new nation state was crucially and fundamentally undercut by something. In seeking to understand what that something was the study has followed Newbigin’s (2014) injunction to re-imagine postcolonial citizenship as something more than the *tabula rasa* that a focus upon the Indian Constitution and charter of Fundamental Rights would suggest. Her notion of the importance of ‘pre-existing debates and social structures’ (Newbigin, 2014: 37) has been borne out in the evidence related earlier, ranging from the reliance upon canonical colonial texts of governance (such as criminal tribes administration manuals) to the habitus of those who were interviewed by the Enquiry Committee and who communicated local governments’ views to it. Yet we are still left with a question: why were the liberty rights of Indians felt to be so easily divisible and a secondary citizenship status for certain communities (of largely un-consulted, un-named individuals) felt so necessary?

That question will reward further study. But one possibility is suggested in the work of Mithi Mukherjee (2010) and it leads us, paradoxically perhaps, back to the Indian Constitution. For although the Constitution is broadly seen as a document of freedom, Mukherjee (2010: 185) draws attention to the fact that its preamble—containing as it does ‘the philosophy of the Constitution framers’—in fact establishes a rather unexpected formulation of such freedom. The preamble, she observes, establishes ‘a network of political categories hierarchically ordered and meant to guide the constitution makers and future legislators in their decisions’ (Mukherjee, 2010: 185). Importantly, justice precedes both liberty and equality in this hierarchy. Such ordering was no mere philosophical fancy either. Part IV of the Constitution, titled the Directive Principles of State Policy, sets out the primacy of justice as the sovereign principle of the Indian legislature and directs the manner of its instantiation in legislative practice. Mukherjee goes on to quote a speech given by Nehru in the *Lok Sabha*, the Indian Parliament, wherein he noted the incommensurability of justice and freedom, adding that ‘it is up to Parliament to remove the contradiction and make fundamental rights *subserve* the Directive Principles of State Policy’ (cited in Mukherjee, 2010: 198, her emphasis).

Mukherjee suggests a continuity and familial resemblance between the 19th-century authoritarian liberalism of figures like JF Stephen and postcolonial conceptions of ‘justice’ as that which gives effect to national priorities above abstract principles. In the new India, she argues, ‘it was the institution of the Parliament—like the British colonial state before it—that would be the primary agent of a new society’ (Mukherjee, 2010: 199). There is certainly merit in this argument. For both, coercion is a legitimate means of government. For Stephen (1874: 166), it finds philosophical support in the prima facie validity of his argument that ‘power precedes liberty—that liberty, from the very nature of things, is dependent upon power; and it is only under the protection of a powerful, well-organised, and intelligent government that any liberty can exist at all’. For the Nehruvian architects of a new India, for whom independence represented something like a permanent state of emergency, the justice of Gandhian ‘uplift’ and broadly based social democracy would require the trump card of coercion. Property rights would need to be qualified in order to achieve land reform. The absolute right to equality would need to be mediated to achieve substantive equality for socially subordinate tribes and classes, and so on.

What was perhaps less anticipated by Indian socialists and claimants of a full and genuine independence was the difficulty that containing these coercive impulses would pose. Moreover, since there never was a complete schism with the past, late-colonial tendencies mixed in possibly unexpected ways with this coercive government. Since at least the 1920s, for example, colonial policy had increasingly sought to effect welfare goals through the machinery of policing, drawing together criminal tribes with other ‘depressed’ classes worthy of state attention and assistance: aboriginals, hill-tribes, untouchables and the like. Thus, the state’s duty of care responsibilities had come to be imagined and enmeshed with structures that were fundamentally coercive and illiberal in nature. Drawing all of this together goes some way to explaining why in 1949–1950, in the immediate shadow of independence, the Enquiry Committee opted for coercion rather than freedom for criminal tribes, recommending the repeal of the Criminal Tribes Act but its immediate replacement with an habitual offender law.

Shortly after, the Indian Parliament did just that, repealing the Act and in its place passing the Habitual Offenders (Control and Reform) Act 1952. Under section 2(1)(c) of that statute members of the formerly notified criminal tribes came to be associated with an alternative but seemingly no less powerful nor stigmatizing moniker, ‘denotified tribes’, or DNTs as they have been referred to in modern Indian parlance. The sequelae of this labelling have included a host of social and economic handicaps as well as a long record of violence and abuse at the hands of higher caste groups and frequently state officials. More recently, there is evidence of certain denotified tribes reversing this relationship of subjection and striking out in increasingly politicized forms of organized violence (Chaturvedi, 2011). As recently as 2007 the United Nations Committee on the Elimination of Racial Discrimination registered its concern ‘that the so-called denotified and nomadic tribes, which were listed for their alleged “criminal tendencies” under the former Criminal Tribes Act (1871), continue to be stigmatized under the Habitual Offenders Act (1952)’ (UNCERD, 2007: 1). In submissions made to the Committee at the time, welfare and advocacy organizations estimated the number of Indian citizens subject to these debilitations to be in the order of 20 million (Resist Initiative International, 2007) and catalogued a range of fundamental rights unavailable to them or diminished by their status (National Network for Human Rights Treaty Monitoring in India, 2007). Clearly, then, understanding the manner in which effective transitions to full rights-bearing citizenship may be achieved is something of profound importance. But if this article has illustrated anything, it is the complexity of the postcolonial condition and the sheer scale of measures visited upon individuals and communities enmeshed in colonial legacies and postcolonial formations of penal power.

## Notes

1. The Criminal Tribes Act Enquiry Committee *Report* (1951) calculated the pre-independence numbers of those residing within the post-independence territorial boundaries of India at just below three million. No estimates exist of numbers in the previously populous (in criminal tribes terms) areas of western Punjab and eastern Bengal, or in other areas ceded to the new state of Pakistan. Verma (2002) puts the figure at 13 million but gives no citation for it nor any description of its estimation.

2. The term ‘local government’ is used in its Indian context to refer to state or provincial governments and legislatures. They were akin to state governments in the USA today.

3. There is now a burgeoning academic literature on the subject. I review most of this in my book *Penal Power and Colonial Rule* (Brown, 2014) which, together with Chaturvedi (2011) and Schwartz (2010) will provide a comprehensive bibliography on the topic. Non-academic texts of some starkly varying quality include Bhadauria (1996), D’Souza (2001), Lalita (1995) and Singh (2010).

4. National Archive of India (NAI), Government of India (GOI) Legislative Proceedings, November 1871, No. 62 (A). These are the closing words of a characterization quoted by JF Stephen on the Bill’s first reading: for the full context see Brown (2014: 107). The words are generally and mistakenly attributed to Stephen. They belong to a Mr Nembhard, the Commissioner of East Berar. There is much confusion in the contemporary Indian non-academic literature on the origins of the Act. D’Souza (2001) is often cited as the best and most important contemporary source, yet his account is replete with gross factual errors and highly unusual historical claims.

5. NAI GOI Legislative Proceedings, November 1871, No. 76(A).

6. NAI GOI Legislative Proceedings, November 1871, No. 58 (A).

7. NAI GOI Legislative Proceedings, November 1871, No. 74 (A).

8. The tenor and divisions of this debate also reflected fundamental differences within 19th-century liberalism that might be roughly characterized as between ‘orthodox’ and ‘authoritarian’ visions of personal freedom and state authority. See generally Brown (2014: ch. 5) and Mehta (1999).

9. NAI GOI (Home—Judicial) July 1872 No. 97 (A).

10. India Office Records, British Library (IOR) GOI L/P&J/6/423 No.1038.

11. For a broader account of the colonial experience of forest dwelling tribes, some of whom, such as the Bhils, were drawn into the control mechanisms of the Criminal Tribes Act, see Skaria (1999).

12. The gendered nature of criminal tribes’ experiences is yet to be explored, though the project had been started at the time of this publication by Jessica Hinchey, at NTU Singapore. Most references I make here are to ‘tribesmen’, but the question of whether tribeswomen should be subject to all the disabilities of registration was a live one during the early years of the Act’s operation. In the Punjab, the issue was first resolved by the Lieutenant Governor, Lepel Griffin, in 1876: women should be registered as well as men, he decided, since: ‘Women are in these professionally criminal classes generally worse than the men, and it is impossible in Indian society to separate the wife from her husband. If they belong to a criminal class or tribe the whole tribe or family must be treated as a unit and not the individual’ (Punjab State Archive, Lahore, Punjab (Home—A) December 1876, No. 1). This decision was later reversed and for the most part only men were subject to registration.

13. For a discussion of the management of ‘predatory’, ‘turbulent’ and ‘criminal’ tribes in areas of military, as opposed to civil, government, see Brown (2014: ch. 4). This discussion also covers the military recruitment of criminal tribes in dedicated local units such as the Meena Battalion (later Deoli Irregular Force) and the Erinpura Irregular Force.

14. IOR MF 1 fiche no. 208, GOI (1877) *Report on the Political Administration of the Rajpootana States, 1876–77*. Calcutta: Foreign Dept Press, p. 4.

15. Ibid., p. 7.

16. NAI GOI (Home—Judicial) February 1880 124–41 (A).

17. Ibid.

18. IOR v/9/192 Legislative Assembly Proceedings, 14 February 1947, p. 624.

19. Bombay Habitual Offenders (Restriction) Act 1947, Madras Restriction of Offenders Act 1948, and Rajasthan Habitual Criminals (Registration and Regulation) Act 1950.

20. NAI GOI Legislative Proceedings, November 1871, No. 57 (A).

21. The story of denotified tribes’ negotiations with the Indian state and their connections with wider social shifts, such as the adivasi movement, is beyond the scope of this article. The focus here is restricted to the years lying roughly between the late 1930s and the early 1950s: the cusp of independence. For the longer view, see generally Chaturvedi (2007), Guha (2015), Schwartz (2010), Singh (2010) and Skaria (1999).

22. IOR L/P&J/5, Home Department Proceedings, October 1918, No. 67.

23. IOR L/P&J/5, Home Department Proceedings, December 1918, No. 111.
